# Early Miocene amber inclusions from Mexico reveal antiquity of mangrove-associated copepods

**DOI:** 10.1038/srep34872

**Published:** 2016-10-12

**Authors:** Rony Huys, Eduardo Suárez-Morales, María de Lourdes Serrano-Sánchez, Elena Centeno-García, Francisco J. Vega

**Affiliations:** 1 Department of Life Sciences, The Natural History Museum, Cromwell Road, London SW7 5BD, United Kingdom; 2 El Colegio de la Frontera Sur (ECOSUR), A.P. 424, 77000 Chetumal, Quintana Roo, Mexico; 3 Posgrado en Ciencias de La Tierra, UNAM, Ciudad Universitaria, Coyoacán, México DF 04510, Mexico; 4 Instituto de Geología, UNAM, Ciudad Universitaria, Coyoacán, México DF 04510, Mexico

## Abstract

Copepods are aquatic microcrustaceans and represent the most abundant metazoans on Earth, outnumbering insects and nematode worms. Their position of numerical world predominance can be attributed to three principal radiation events, *i.e.* their major habitat shift into the marine plankton, the colonization of freshwater and semiterrestrial environments, and the evolution of parasitism. Their variety of life strategies has generated an incredible morphological plasticity and disparity in body form and shape that are arguably unrivalled among the Crustacea. Although their chitinous exoskeleton is largely resistant to chemical degradation copepods are exceedingly scarce in the geological record with limited body fossil evidence being available for only three of the eight currently recognized orders. The preservation of aquatic arthropods in amber is unusual but offers a unique insight into ancient subtropical and tropical ecosystems. Here we report the first discovery of amber-preserved harpacticoid copepods, represented by ten putative species belonging to five families, based on Early Miocene (22.8 million years ago) samples from Chiapas, southeast Mexico. Their close resemblance to Recent mangrove-associated copepods highlights the antiquity of the specialized harpacticoid fauna living in this habitat. With the taxa reported herein, the Mexican amber holds the greatest diversity of fossil copepods worldwide.

Copepods are among the most speciose and morphologically diverse groups of crustaceans, encompassing 236 families and roughly 13,970 described species. In terms of individuals they outnumber every other group of metazoans on Earth, including the hyperabundant insects and nematode worms^1^. Current evidence suggests that copepods originated from the marine hyperbenthic habitat, however, their position of numerical world predominance can be attributed to three principal, recurrent, radiation events, *i.e.* their major habitat shift into the marine plankton, the colonization of freshwater and semiterrestrial environments and the evolution of parasitism. In the pelagic realm, the largest biome on the planet, copepods are the dominant members of the holozooplankton, both numerically and in terms of biomass. In addition to life strategies that encompass free-living, substrate-associated, and interstitial habits, copepods also have extensive impacts in their role as associates or parasites of the majority of aquatic metazoan phyla, from sponges to chordates, including reptiles and marine mammals. This variety of life strategies has generated an incredible morphological plasticity and disparity in body form and shape that are arguably unrivalled among the Crustacea^2^. Despite widespread interest in this group, phylogenetic relationships among copepods remain unsettled at many taxonomic levels. Complicating interpretation of these lineages is the estimated 459 million years (Ma) since the origin of the Copepoda^3^ coupled with the group’s morphological diversity.

Although their exoskeleton is mainly composed of chitin and sclerotized structures and thus largely resistant to chemical degradation, copepods are exceedingly scarce in the geological record. Eight orders of copepods are currently recognized^2^ but limited body fossil evidence is only available for three of them, *i.e.* Harpacticoida, Cyclopoida and Siphonostomatoida (Fig. 1). Fossil harpacticoids and cyclopoids were first discovered from the Miocene Barstow Formation in the Mojave Desert in southern California^4^,^5^. While the former were assigned to the present-day genus *Cletocamptus* Shmankewich, 1875, the cyclopoids remained unidentified. However, recent collections from the same formation resulted in numerous exceptionally preserved fossils, including adult and larval stages, of a new species of the extant genus *Apocyclops* Lindberg, 1942^6^. Additional harpacticoid fossils were reported from a Pleistocene sedimentary lake deposit associated with borate minerals in Argentina^5^ but this record is merely anecdotal evidence since no descriptive information was provided. The only fossil member of the Siphonostomatoida was discovered on the gills of a fossil teleost fish, *Cladocyclus gardneri* Agassiz, 1841, preserved in calcareous nodules in the Lower Cretaceous (110–120 Ma) Santana Formation in northeastern Brazil’s Araripe Basin^7^,^8^. The recent discovery of harpacticoid fragments in a bitumen clast of late Carboniferous age (*ca.* 303 Ma) in Eastern Oman extended the fossil record of copepods by some 188 Ma and provided the oldest confirmed body fossil evidence for the group^3^. Middle–Late Cambrian crustacean mandibular gnathobases were discovered from the Deadwood Formation (488–510 Ma) in Saskatchewan and Alberta (Canada)^9^ and from the Nolichucky Shale (495–500 Ma) in Tennessee (USA)^10^. Although it has been conjectured that the relatively large mandibles could belong to a stem or crown group copepod of centimetric size^9^ the claimed similarity, based on isometric growth and the presence of a dorsal seta, with extant members of the group appears unconvincing in the absence of other identifiable body parts. Most gnathal edges from the Canadian deposits possess filamentous structures unknown in modern copepods, while the spinous fragments from the Nolichucky Shale display a radically different morphology not previously observed in copepod mandibles. Despite the considerable variability in the fine-scale morphology observed in the “mandibular teeth” from both geological formations it has also been suggested that they represent closely related taxa, perhaps a single species^10^. A potential copepodan affinity was proposed for two unnamed fossils from the Lower Ordivician of Öland, Sweden^11^. Although the number of thoracic somites (each bearing paired biramous legs) in these fossils is reminiscent of the copepodan groundpattern, the presence of stalked eyes and the larval morphology precludes their placement in that group. The proposal that the predominantly Paleozoic Cycloidea were copepods (N. D. L. Clark, unpublished PhD dissertation, University of Glasgow, 1989) is equally indefensible since cycloideans display multisegmented antennae, uniramous “walking limbs” and lack intercoxal sclerites. Trace fossil evidence of parasitic copepods associated with vertebrate and invertebrate hosts has recently been put forward for consideration^12^, however in both cases there is no direct indication for ancient symbiosis. Exocysts found on the surface of fossil echinoid tests, referred to as the ichnotaxon *Castexia douvillei* Mercier, 1936, and the so-called Halloween pumpkin-mask cysts known from both echinoids and crinoids, have been suggested to be treatable as Jurassic trace fossils of copepods in central Europe, Israel and around the Caspian Sea^12^. Lesions apparent on Devonian sarcopterygian and placoderm fish in Latvia, Estonia and western Russia have been tentatively attributed to infestation by ectoparasites, including copepods^13^, however, since other causative agents are conceivable the association with copepods cannot be confirmed^14^. Subfossil copepod eggs^15^,^16^,^17^,^18^,^19^, egg envelopes^20^,^21^ and spermatophores^22^,^23^ have been reported from Neogene and Quaternary deposits and some acritarchs from the Lower Cretaceous in Brazil^24^,^25^,^26^ and Florida^27^ have been interpreted as copepod eggs.

One of the most auspicious fossiliferous sources of extinct arthropods is amber, a fossilized resin that was produced from the trunks and roots of certain trees. Although resins are produced by a wide range of flowering plants and by conifers, only two plant genera exudate it in a form that resists biological, chemical and physical degradation and are responsible for most of the fossiliferous amber deposits known today. It occurs in many areas of the globe, representing approximately 170 amber deposits, some dating from as early as the Late Carboniferous (*ca.* 300–320 Ma)^28^. Amber is a unique preservational mode since, more than any other type of fossilization, it maintains trapped plants and animals in their three-dimensional form, a phenomenon already noted by the Roman natural philosopher Pliny the Elder. Although the oldest records of arthropods preserved in fossilized resin date from the Late Triassic (*ca.* 230 Ma)^29^ their occurrence in amber, almost exclusively from the Cretaceous and Cenozoic, is widely regarded to be a result of the production and preservation of large amounts of tree resin beginning *ca.* 130 Ma ago. There is a vast volume of published literature about the diversity of insects and other terrestrial arthropods preserved in amber^30^,^31^ but very little is known about crustaceans in general. Except for a few recent reports documenting fossil tanaidaceans^32^,^33^,^34^ and decapods^35^ as bioinclusions, marine crustaceans appear to be remarkably scarce or practically absent in amber and the authenticity of copepods recorded in Cretaceous amber^34^,^36^ requires verification^3^.

The amber from Campo La Granja mines in Chiapas, southeast Mexico represents one of the most significant Cenozoic deposits in terms of its biological inclusions, comprising a unique mixture of terrestrial, freshwater and brackish water arthropod fauna^37^. Crustacean groups (ostracods, tanaidaceans, amphipods and isopods) not usually found as inclusions in amber are relatively common in this area and are found associated with both freshwater and terrestrial insects. This mixture of ecologically diverse groups suggests an allochtonous assemblage deposited in small shallow tidal-flat ponds adjacent to a mangrove-like shore^37^. Here we report a unique occurrence of mangrove-associated copepods of the order Harpacticoida preserved in a collection of several amber pieces collected in the Campo La Granja mines in Chiapas State, Mexico. The inclusions not only present the first confirmed records of copepods in amber but also provide the greatest diversity of fossil copepods worldwide and new paleoecological data on the Early Miocene habitat in Middle America.

## Geological setting

**Figure 2:**
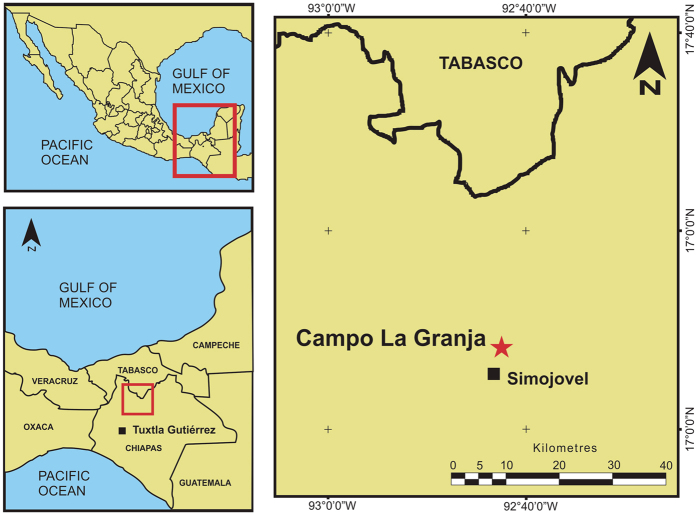
Location. Map showing relative position of Campo La Granja mines, north of Simojovel de Allende town, Chiapas, Mexico. Map drawn with CorelDRAW Graphics Suite X7 (version 17) (http://www.coreldraw.com/gb/product/graphic-design-software/).

**Figure 3:**
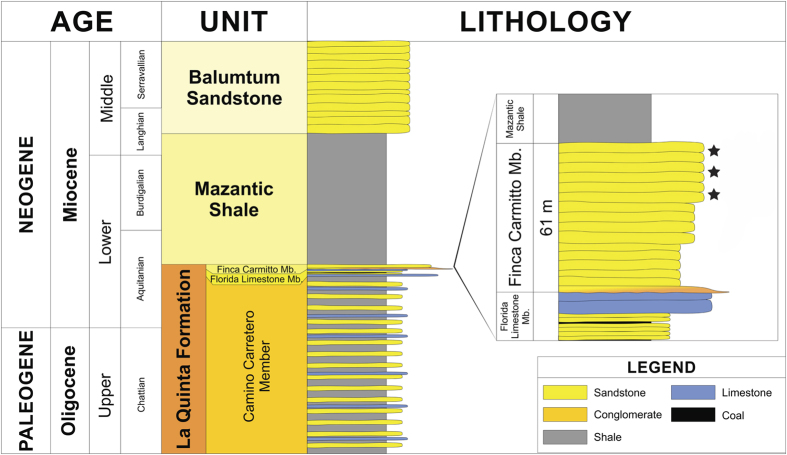
Stratigraphic position of Campo La Granja mines, located within the upper member (Finca Carmitto Member) of the Lower Miocene sandstone of the La Quinta Formation, Chiapas. Samples examined in this study indicated by asterisks. Modified from^38^ (see also R. C. Allison, unpublished PhD dissertation, University of California, Berkeley, 1967).

The Campo La Granja amber mines are located in the northern sector of Chiapas State, Southern Mexico, 800 m northeast of the town of Simojovel de Allende (17°08′48.35″N, 92°42′30.50″W) (Fig. 2). Chiapas amber is primarily found in three lithostratigraphic units which are exposed in the Sierra Madre del Sur, from the north edge of the central depression (Totolapa) to Palenque, near the Gulf Coast of Tabasco, known as (from base to top): La Quinta Formation, Mazantic Shale and Balumtum Sandstone^38^,^39^,^40^,^41^ (see also R. C. Allison, unpublished PhD dissertation, University of California, Berkeley, 1967). The Campo La Granja mine tunnels were excavated in outcrops of the upper portion (Finca Carmitto Member) of the La Quinta Formation, a 884 m thick sequence that crops out northeast of the La Esperanza syncline and southwest of the Simojovel syncline^39^ (Fig. 3). Based on strontium isotope ratios (^87^Sr/^86^Sr) in gastropod shells and biostratigraphic data of corals, molluscs, microfossils (nannoplankton) strata in the Finca Carmitto Member were dated as Aquitanian (Early Miocene) in age (22.8 Ma)^37^,^38^,^40^. Amber pieces recovered by local miners from the Campo La Granja mines range in size from less than one centimeter up to 60 cm in length; those examined in this study varied in size from small (3 × 3 × 0.5 cm) to medium-sized (8 × 5 × 2 cm). Most have clear layers of diverse thickness, from less than one to four millimeters, representing individual resin flows, separated by thin layers of sand. Many of the amber layers are themselves stratified with quartz/rich sandstone similar to that of the surrounding rock matrix of the Finca Carmitto Member. Sandstone interlayered with the amber horizons is fine to medium, well-sorted and grain-supported. Sand grains are mostly mono-mineral (quartz abundant) and subangular to sub-rounded in shape, with few well rounded clasts, showing a low level of compaction. Sandstone layers also contain abundant feldspar grains consisting of plagioclase, pertite and orthoclase. Detrital mica is very abundant and primarily represented by biotite with some muscovite and chlorite, suggesting low influence of chemical weathering at the source area. Quartz grains have apatite inclusions, and heavy minerals such as zircon and apatite are abundant. Percentages of quartz and feldspar suggest that the sandstone is an arkose to subquartzarenite. Its composition indicates a crystalline source rock, such as high-grade metamorphic or granitic rocks^37^.

The botanical source of Chiapas amber has recently been determined as being the result of resinous exudates produced during the late Oligocene to early Miocene by two species of the leguminose tree genus *Hymenaea* L. (family Fabaceae), *H. mexicana* Poinar & Brown, 2002 and *H. allendis* Calvillo-Canadell, Cevallos-Ferriz & Rico-Arce^42^. Paleontological evidence indicates that *Hymenaea* communities developed near the ancient coast in estuarine habitats resembling modern mangrove forests^38^,^42^. During Early Miocene times, the area of Simojovel was located on the coast of the Gulf of Mexico and biological inclusions such as aquatic insects, grapsoidean crabs and copepods reinforce the presence of an ancient mangrove environment^37^,^38^. Micro-cross-bedding and other sedimentary structures suggest a tidal influence in an estuarine environment that may have transported meiofaunal crustaceans into small localized ponds where resin was deposited. The presence of organic debris, pyrite, occasional pyritized arthropods and psychodid flies^43^ suggests reducing conditions and low pH levels prevailed in the stagnant water of the ponds, possibly caused by decomposing soil organic matter^37^. Additional evidence in support of an estuarine fluvial paleoenvironment is provided by the presence of both estuarine and freshwater taxa in the amber pieces. The presence of obligatory aquatic crustaceans (ostracods, copepods) preserved between the amber layers suggests that the amber must have been deposited under water^37^,^44^. Deformation features observed in some of the amber pieces examined suggest that the resin was not always fully solidified in between flows^37^. Tidal influence could explain how resin bodies acquired sand grains on their surfaces in between successive resin flows.

## Results

**Figure 4:**
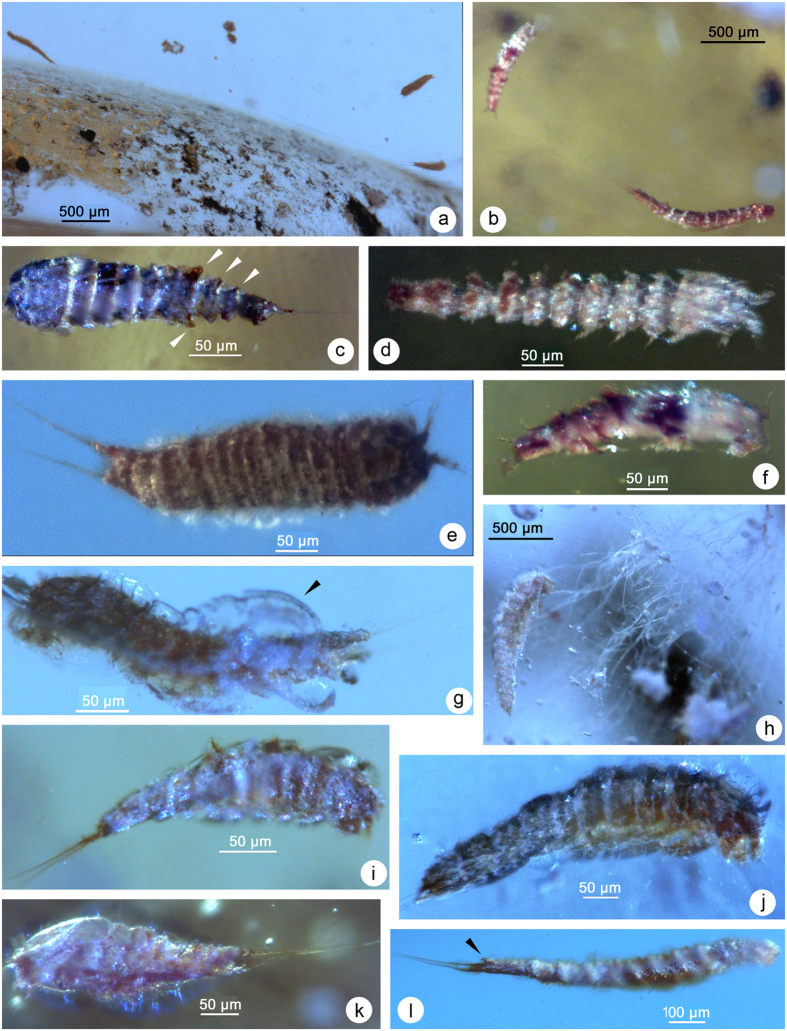
Early Miocene harpacticoid copepods from Campo La Granja amber mines, Chiapas. (**a**) overview at low magnification showing *in situ* position of IHNFG–5312/Cop1–3. (**b**) *in situ* view of IHNFG–5315/Cop1–2, showing *Darcythompsonia* sp. (♀) [bottom right] and unidentified copepod (top left]. (**c**) Laophontidae sp. (♀) (IHNFG–4948/Cop1); arrows indicating strongly developed pleurotergites of genital double-somite and postgenital abdominal somites. (**d**) Cletodidae sp. (♀) (IHNFG–5315/Cop3). (**e**) Canthocamptidae sp. (*Cletocamptus* sp.) (♀) (IHNFG–5312/Cop2). (**f**) Unidentified copepodid, family unconfirmed (IHNFG–5315/Cop1). (**g**) Canthocamptidae sp. (possibly *Cletocamptus* sp.) (♀) in process of moulting (IHNFG–5313/Cop1); exuvium of preceding copepodid V stage arrowed. (**h**) *Enhydrosoma* sp. 1 (♀), Cletodidae (IHNFG–5314/Cop1); note large body size (compare Figs 4j and 5b). (**i**) Unidentified copepodid, family unconfirmed (IHNFG–5314/Cop2). (**j**) *Enhydrosoma* sp. 2 (♀), Cletodidae (IHNFG–5314/Cop3); note short caudal rami (compare Fig. 5b). (**k**) Unidentified copepodid, family unconfirmed (IHNFG–5311/Cop1. (**l**) *Darcythompsonia* sp. (♂), Darcythompsoniidae (IHNFG–5312/Cop1); arrow indicating raised bifid anal operculum.

**Figure 5:**
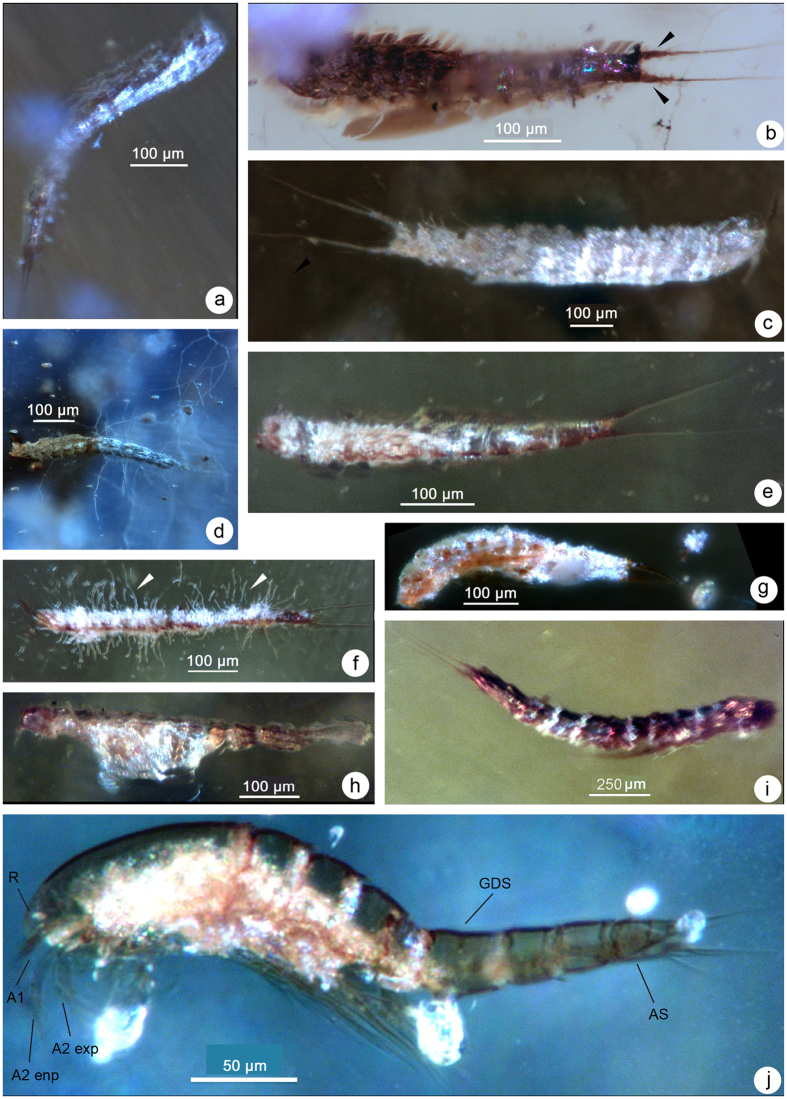
Early Miocene harpacticoid copepods from Campo La Granja amber mines, Chiapas. (**a**) *Leptocaris* sp. (sex undetermined), Darcythompsoniidae (IHNFG–5312/Cop4). (**b**) *Enhydrosoma* sp. 3, Cletodidae (IHNFG–5314/Cop4); elongate, cylindrical caudal rami arrowed (compare Fig. 4h,j). (**c**) *Leptocaris* sp. (IHNFG–5315/Cop4). (**d**) *Leptocaris* sp. (♀), with surrounding hyphal growth (IHNFG–5314/Cop5). (**e**) Ectinosomatidae sp. (?) (IHNFG–5315/Cop5). (**f**) *Leptocaris* sp. (IHNFG–5315/Cop6); fungal hyphae attached to exoskeleton arrowed. (**g**) Unidentified copepodid, family unconfirmed (IHNFG–5311/Cop2). (**h**) *Leptocaris* sp. (IHNFG–5315/Cop7). (**i**) *Darcythompsonia* sp. (♀) (IHNFG–5315/Cop2). (**j**) *Halectinosoma* sp. or *Pseudobradya* sp. (♀), Ectinosomatidae (IHNFG–4899/Cop1); A1 = antennule, A2 enp = antennary exopod, A2 enp = antennary endopod, GDS = genital double-somite, AS = anal somite.

**Figure 6:**
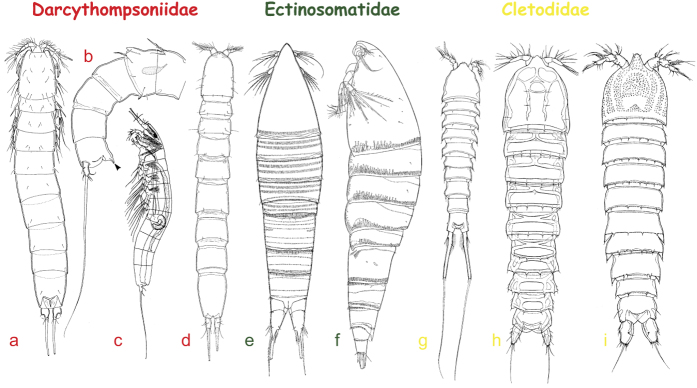
Representative present-day members of principal harpacticoid families preserved in Chiapas amber inclusions. (**a–d**) Darcythompsoniidae; (**e,f**) Ectinosomatidae; (**g–i**) Cletodidae. (**a,d,e,g–i**) dorsal view; (**b,c,f**) lateral view. (**a**) *Darcythompsonia fairliensis* (T. Scott, 1899) (♀). (**b**) *D. fairliensis* (♂), urosome (arrow indicating bifid, dorsal spinous process on anal somite). (**c**) *Horsiella brevicornis* (von Douwe, 1904) (♂). (**d**) *Leptocaris pori* Lang, 1965 (♀). (**e**) *Halectinosoma ornatum* Lang, 1965 (♀). (**f**) *Pseudobradya pulchera* Lang, 1965 (♀). (**g**) *Stylicletodes longicaudatus* (Brady, 1880). (**h**) *Enhydrosoma hopkinsi* Lang, 1965. (**i**) *Cletodes hartmannae* Lang, 1965.

A total of 69 nearly complete copepods were observed in 14 amber pieces from sandstones of the upper part of La Quinta Formation, Chiapas, Mexico (Table 1). All specimens were generally well preserved and could confidently be attributed to the order Harpacticoida. No calanoids or cyclopoids were present among the inclusions. Most individuals were found in clusters (2, 4, 9), usually next to a layer of sandstone and organic matter. The harpacticoids were found together with other crustaceans as syninclusions such as estuarine ostracods, isopods and amphipods. Ten putative species, representing at least six genera and five families in the Harpacticoida, were identified based primarily on general body shape, antennulary length and caudal ramus morphology (Figs 4 and 5; Table 1). Except for 21 juvenile (copepodids) or incomplete specimens, which proved indeterminable, most copepods resemble present-day representatives (Fig. 6).

The Darcythompsoniidae, represented by two species, was by far the most common family among the amber inclusions (Figs 4b,l and 5a,c,d,f,h,i; Table 1). Members of this group are typically elongate and cylindrical or vermiform, lacking a marked distinction between the prosome and urosome (Fig. 6a–d)^45^,^46^,^47^,^48^. Other diagnostic characters include the short caudal rami with only one well developed terminal seta (V), reduced oral appendages and short antennules. The majority of the specimens can be attributed to the *brevicornis*-species group (=*Horsiella* Gurney, 1920) of *Leptocaris* T. Scott, 1899 (Fig. 5a,c,d,f,h) and mostly vary in size between 430 μm (Fig. 5d) and 485 μm (Fig. 5h). One specimen (Fig. 5c) has a more robust appearance, is distinctly larger (870 μm) and may represent a different species. Other darcythompsoniids include two females (Figs 4b and 5i) and one male (Fig. 4l) of the genus *Darcythompsonia* T. Scott, 1906 which accommodates species characterized by a larger body size. There is a remarkable similarity in size between the Chiapas specimens (♀: 1,090 μm; ♂: 850 μm) of *Darcythompsonia* sp. and populations of *D. fairliensis* (T. Scott, 1899) reported previously from Mexico (♀: 1,155 μm)^47^ and the Galápagos (♀: 1,100–1,200 μm; ♂: 850 μm)^48^, the European populations of this species being considerably larger (♀: 1,500–1,730 μm)^49^.

A single female (Fig. 5j; 335 μm) could be unequivocally attributed to the family Ectinosomatidae while another, more slender, specimen (Fig. 5e; 470 μm) may also belong to this group. Non-interstitial ectinosomatids have a characteristically fusiform body with a subtriangular cephalothorax, short antennules, large antennary exopods (almost as long as the corresponding endopods) and a medially cleft anal somite (Fig. 6e,f)^46^,^49^. The yellowish brown colour of the integument in the preserved specimen (Fig. 5j) most likely indicates that it belongs to *Pseudobradya* Sars, 1904 or *Halectinosoma* Vervoort, 1962, the only two extant genera that accommodate representatives displaying this feature^49^. Optical cross-sections (Fig. 5j) showed that the brown pigment is restricted to the thick laminated procuticle, being absent from the overlying non-chitinous epicuticle and the underlying tissues. Its association with the procuticular chitin rods, which are set into a protein matrix, probably ensured its long term preservation.

At least three putative species of the cletodid genus *Enhydrosoma* Boeck, 1873 (all from amber fragment IHNFG–5314) could be identified based on differences in caudal ramus length and body size (Figs 4h,j and 5b). *Enhydrosoma* sp. 1 (Fig. 4h) is distinctly larger (1,290 μm) than the other two species (sp. 2: 530 μm; sp. 3: 485 μm). *Enhydrosoma* sp. 3 (Fig. 5b) has elongate, cylindrical caudal rami *vs.* short, conical caudal rami in the other species (Fig. 4h,j). A fourth specimen (Fig. 4d; 440 μm) from fragment IHNFG–5315 is probably also attributable to the Cletodidae. Members of this family tend to be heavily chitinized and stoutly built (Fig. 6g–i). Their bodies are cylindrical, lacking a conspicuous boundary between prosome and urosome but often displaying marked constrictions between individual somites (Fig. 4d, *cf.* 6g). Cletodid specimens assume a typically arched shape when preserved (Fig. 4h,j).

A small specimen from fragment IHNFG–4948, measuring around 260 μm in length, was identified as a member of the family Laophontidae based on the strongly developed pleurotergites of the genital double-somite and postgenital abdominal somites (Fig. 4c). Three specimens (Fig. 4e,g; 325–390 μm) from three different fragments (Table 1) are provisionally attributed to the genus *Cletocamptus* Shmankewich, 1875, based on their general body facies, length of antennules and caudal ramus shape.

Direct sexual determination was possible in a few cases. Females could usually be identified by the presence of a genital double-somite (Fig. 5j) or the absence of geniculate antennules (Fig. 4e)^46^. One of two specimens of *Darcythompsonia* sp. (IHNFG–5312/Cop1) could be positively determined as a male based on the sexually dimorphic spinous anal operculum (Fig. 4l). This character is unique to extant members of the genus (Fig. 6b)^45^,^47^. Ovigerous females or mate guarding were not observed. In several specimens the exoskeleton was covered by filamentous fungal hyphae (Figs 4h and 5d,f).

All specimens are regarded as body fossils and must have been trapped in the resin alive. They do not represent exuviae which could easily have been wind transported after desiccation from coastal areas to resiniferous forests. Findings of several copepods within a single piece of amber (Fig. 4a) make the hypothesis of wind-blown dead organisms very improbable. The discovery of an adult copepod (possibly *Cletocamptus* sp.; Fig. 4g) in the act of emerging from its old exoskeleton has not been documented before in the fossil record and may have been caused by the stress of struggling against the sticky resin. Some trapped specimens produced distortion (struggling marks) in the resin flows (Fig. 5b).

## Discussion

The primarily benthic Harpacticoida currently includes about 4,675 valid species placed in 652 genera and 58 families and is arguably the order that has undergone the greatest diversification in copepod evolution. While some display an extraordinary range of intraspecific variability, inability to recognize cryptic or sibling species using traditional morphological analyses has led to an underestimation of the true diversity of this group and an overestimation of potential for long-distance dispersal in putatively cosmopolitan species. During their extensive ecological radiation since at least Carboniferous times^3^ harpacticoids succeeded in colonizing virtually all aquatic habitats (marine, brackish, and fresh water) and became ubiquitous in the marine environment, occurring from tidal pools to the hadal zone of the deep sea. Despite this evolutionary success confirmed fossils of harpacticoid copepods are extremely rare and were previously known from only three countries in the world. Fossilized specimens assignable to *Cletocamptus* (Canthocamptidae *incertae sedis*) have been found in sedimentary lake deposits associated with Boron minerals in the Barstow Formation in the Mojave Desert in Southern California, dating to the Early-Middle Miocene (13.4–19.3 Ma)^4^. The body fossils were considered similar to an extant species, *C. albuquerquensis* (Herrick, 1894) and subsequently attributed to *C. retrogressus* Schmankewitsch, 1875^5^ but could not be identified with confidence due to lack of preservation of key diagnostic characters. Additional fossils were reported from a similar Pleistocene lake deposit in Argentina but were classified only to ordinal level without further information^5^. Finally, evidence supporting Paleozoic invasion of the continental waters in Pangaea by harpacticoid copepods was recently provided by fossilized fragments of cephalothoracic appendages from a bitumen clast in a glacial diamictite in eastern Oman^3^. The fragments were of late Carboniferous age (*ca.* 303 Ma) and assignable to the family Canthocamptidae. The subfossil record of harpacticoids is equally meagre, consisting of a single specimen of *Enhydrosoma gariene* Gurney, 1930 (Cletodidae) from a Neolithic excavation site in Kent in southern England^50^ and a few spermatophores from subboreal peat deposits in Germany which were attributed to *Canthocamptus* sp.^22^,^23^.

Although amber acts as a natural embedding agent that preserves trapped fragile organisms more completely than any other type of fossilization, no confirmed records of copepod inclusions have been documented before. Possible Cretaceous copepods have been reported from Canada^36^ and Spain^34^ but both records are highly contentious. The specimen labelled as a potential copepod from Chemawinite or Canadian amber^36^ was not figured or described and could not be traced in the collections of the Museum of Comparative Zoology at Harvard University^3^. A copepod-like individual caught at the tip of the antennule of the fossil tanaidacean *Alavatanais carabe* Vonk & Schram^34^ was reported from Early Cretaceous (Aptian-Albian) Álava amber in northern Spain but the accompanying photograph does not provide any convincing evidence to substantiate the authenticity of this claim. The present discovery of harpacticoid inclusions in Mexican amber represents not only the first formal record of the group from fossiliferous resin deposits but also produced the greatest diversity of fossil copepods worldwide so far.

Within mangrove sediments, as in most estuarine habitats, the numerically dominant metazoans are the meiofauna with nematodes and harpacticoids usually constituting over 90% of the hard-bodied component of this faunal assemblage. Analyses of community structure of these taxa revealed that both are concentrated within the surface layers of the sediment, however communities associated with decomposing leaves are distinct from those associated with the sediment surface^51^. There are no nematodes which are found exclusively on decaying leaves but some harpacticoids have an obvious predilection for the peculiar niche presented by mangrove litter. Leaves falling off mangrove trees onto the sediment surface become nutritious due to the microbial enrichment process during decomposition and offer a pristine habitat for colonization by meiofaunal organisms. Various studies in fringe mangrove forests in Florida^52^, lagoonal mangal habitats in Brazil^45^,^53^ and mangrove forests in peninsular Malaysia^51^,^54^ have suggested that darcythompsoniids are the most typical harpacticoids of mangrove litter systems and play an important role in their degradation. Members of this group possess a very prominently developed labrum and a robust mandibular gnathobase with very powerful associated musculature while the postmandibular cephalic limbs are typically reduced or non-functional. Such a mouthpart design suggests that these animals feed by gnawing at, or scraping, a soft flat substrate such as leaves, and mechanically crushing their food^54^. Although it has been conjectured that they may feed directly on the cellular material of the leaves^54^, gut contents analysis indicates that they are capable of feeding on detritus and fungi associated with decomposing leaves^45^,^53^. Unlike other harpacticoids members of the Darcythompsoniidae do not brood their eggs in egg-sacs^2^ but deposit them directly onto (or into) the leaf litter where the entire life cycle is completed and appears to be synchronized with the decay process in the leaves. The predominance of darcythompsoniids in the Chiapas amber samples suggests that this intimate relationship with the decomposition cycle was probably already established during Miocene times. Species of the families Ectinosomatidae and Cletodidae are typically abundant in the upper few mm of the muddy substrate surrounding decaying leaves on the mangrove floor but are rarely encountered in the leaf communities^51^. Some *Cletocamptus* species are typical inhabitants of mangrove environments but do not appear to have a preference for a particular microhabitat^53^,^55^. In general, the strong congruence between Miocene and Recent mangrove-associated copepod communities testifies to the antiquity of the specialized harpacticoid fauna living in this habitat.

Bacteria and fungi play an important role in the degradation of mangrove leaf litter and extant harpacticoid copepods living in this microhabitat are often covered by their epizoic equivalents (R.H., unpublished data). However, recent experiments designed to investigate the embedding of aquatic organisms in modern tree resin in a swamp forest environment showed that filiform bacteria and fungi may continue to grow inside the resinous exudate as long as it had not solidified^44^. The randomly orientated filamentous fungi observed on the exoskeleton of several amber-embedded harpacticoids in the Chiapas material is likely the result of a secondary accumulation of hyphae over the first couple of weeks following the initial entrapment in the resin.

The widespread assumption that resin dried exclusively on the bark of trees, in conjunction with its extreme hydrophobicity, was until recently difficult to reconcile with the fact that the best-preserved fossils of aquatic arthropods are found in amber^44^. An experimental study based on modern tree resin demonstrated that resinous exudates flowing down the tree trunk into water split up into three fractions, *i.e.* a thin film at the water surface, small resin pieces hanging at the water surface but retaining contact with the tree trunk, and large subaquatic resin outflows which are too heavy to remain at the water surface^44^. The latter do not solidify as long as they are covered with water and are the most likely fraction to trap aquatic organisms. Among the meiofaunal taxa, copepods are predestined for embedding since their high motility exposes them to a higher probability of encountering and becoming attached to subaquatic resin flows than their soft-bodied counterparts. All fossils reported here were found inside the amber pieces and completely surrounded by the fossilized resin. None of the copepods was located in microfissures or at the external surface of the amber fragments, suggesting they are genuine inclusions that were trapped and subsequently enclosed by the resin when it was in its liquid stage and are not the result of past-Early Miocene contamination. During the Early Miocene the area of Campo La Granja was situated near the coast of the Gulf of Mexico. Tides probably played an important role in transporting decaying mangrove leaves and their associated fauna to the legume tree environment of resin deposition, probably small, shallow ponds adjacent to the mangrove forests. Since changing water levels were required for solidification of the subaquatic resin it probably remained fluid until the ponds dried up. Marine microfossils from amber are extremely rare^32^,^34^. Diverse marine diatoms as well as radiolarians, foraminiferans, siliceous sponge spicules and spines of larval echinoderms were recently discovered in Late Albian and Early Cenomanian (*ca.* 100 Ma) amber fragments in southwestern France^56^. The sources of the mid-Cretaceous amber were mixed coastal forests dominated by the conifer families Araucariaceae and Cheirolepidiaceae, similar to the modern *Araucaria columnaria* forests in New Caledonia. However, while it was suggested that wind, spray, and possibly high tides may have transported shells and microremnants of marine organisms from the shore or the sea onto the resin flows of these woods^56^, it is unlikely that wind-aided introduction was a primary mechanism in the transportation of the Chiapas fossils and their subsequent engulfment by resinous exudates.

A major problem with arthropods preserved in amber is establishing their precise taxonomic identity. Various Holocene and Tertiary insect specimens, mostly from amber inclusions, have been labelled as morphologically indistinguishable from, and consequently conspecific, with Recent species^57^. The age of these fossils ranges from 10,000-yr-old Ice Age specimens to amber and sediment fossils dated between 15 and 45 Ma but in many cases their conspecificity with extant species has not been subjected to critical scrutiny. Although insect species can exist for several million years, the average duration has been suggested to range from 2–10 Ma depending on the authority^30^,^58^. However, a recent study of the minute archostematan beetle *Micromalthus debilis* LeConte, 1878 from 20–30 Ma-old Dominican amber showed that, once environmental conditions remain stable, evolutionary stasis may persist for even longer periods and result in species continuity since the Miocene epoch. Alternatively, when morphological stasis continues after a speciation event, resulting species may continue to diverge genetically in the absence of morphological differentiation, producing cryptic species. Analysis of five gene regions in samples of the polymorphic meiobenthic harpacticoid copepod *Cletocamptus deitersi* (Richard, 1897) from four localities in North America revealed four extremely differentiated molecular lineages with unalignable nuclear intergenic spacers and mitochondrial uncorrected divergences reaching levels (25–36%) that are substantially greater than those reported previously for congeneric species in other crustaceans^59^. Retrospective analysis showed that morphological differences among the major lineages were subtle but congruent with the patterns of genetic differentiation, corroborating the presence of cryptic species^60^. A molecular clock applied to the cytochrome oxidase subunit I data suggested that these lineages diverged in the Miocene, consistent with the fossil record of a North American *Cletocamptus* from the same epoch^4^. The darcythompsoniid species entombed in the Chiapas amber likewise show a striking similarity to their present-day Mexican representatives of the family. *Leptocaris* sp. (Fig. 5i) is potentially conspecific with *L. stromatolicolus* which was originally described from stromatolites in two closed evaporitic lakes and a marsh in the Cuatro Ciénegas basin in central Coahuila^61^ while *Darcythompsonia* sp. (Fig. 4l) is morphologically similar to *D. fairliensis* (T. Scott, 1899), recorded from the coastal Ensenada del Pabellon lagoon in the south-eastern Gulf of California^47^ but also from Europe^49^ and the Galápagos Islands^48^. Pending the arrival of molecular sequence data of present-day geographically separated populations it remains an open question whether the Mexican darcythompsoniids represent another case of deep evolutionary lineages that have undergone long-term morphological stasis. Consequently, in the absence of such vital data we have elected not to formally name the Chiapas harpacticoids.

## Material and Methods

### Sample processing

Amber samples were cut with a diamond saw, polished with Brasso metal polish and examined with a SZH Olympus™ high-end stereo microscope. Specimens were photographed with an Axio Zoom.V16 Zeiss fluorescence stereo zoom microscope, using a PlanNeoFluar Z 2.3x/0.57 FWD 10.6 mm objective. Amber fragments and fossils are deposited in the Museo de Paleontología “Eliseo Palacios Aguilera” (Secretaría de Medio Ambiente e Historia Natural), Tuxtla Gutiérrez, Chiapas, Mexico, under the acronym IHNFG (Instituto de Historia Natural, Fósil Geográfico). Copepod specimens are labelled after the amber piece in which they were enclosed and bear the suffix “/Cop#”.

## Additional Information

**How to cite this article**: Huys, R. *et al*. Early Miocene amber inclusions from Mexico reveal antiquity of mangrove-associated copepods. *Sci. Rep.* **6**, 34872; doi: 10.1038/srep34872 (2016).

## Figures and Tables

**Figure 1:**
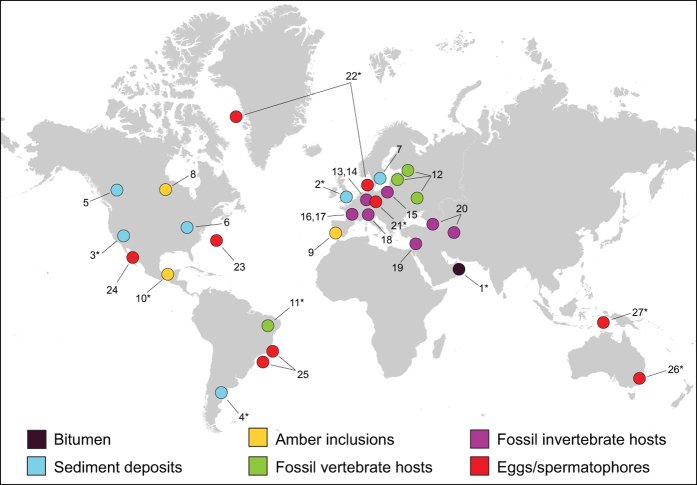
Localities of all confirmed (indicated by *) and unconfirmed records of (sub)fossil Copepoda. **(1)** Oman, Al Khlata Formation, Late Carboniferous (c. 303 Ma), harpacticoids^3^; **(2)** United Kingdom, Kent, Holocene (Neolithic), harpacticoids^50^; **(3)** USA, California, Early-Middle Miocene (13.4–19.3 Ma), harpacticoids and cyclopoids^4^,^5^,^6^,^62^,^63^; **(4)** Argentina, Pleistocene, harpacticoids^5^; **(5)** Canada, Saskatchewan and Alberta, Middle-Late Cambrian (488–510 Ma)^9^; **(6)** USA, Tennessee, Middle-Late Cambrian (495–500 Ma)^10^; **(7)** Sweden, Öland, Early Ordovician (477–485 Ma)^11^; **(8)** Canada, Manitoba, Late Cretaceous (60–80 Ma)^36^; **(9)** Spain, Álava, Early Cretaceous (100–120 Ma)^34^; **(10)** Mexico, Chiapas, Early Miocene (22.8 Ma), harpacticoids (this study); **(11)** Brazil, Ceará, Early Cretaceous (110–120 Ma) siphonostomatoids^7^,^8^; **(12)** Estonia, Latvia and European Russia, Middle–Late Devonian (358.9–387.7 Ma)^13^; (**13)** Germany, Early Jurassic (190.8–199.3 Ma), crinoid cysts; **(14)** Germany, Late Jurassic (152.1–157.3 Ma), echinoid cysts; **(15)** Poland, Middle (163.5–166.1 Ma) and Late Jurassic (152.1–163.5 Ma), echinoid cysts^12^; **(16)** France, Middle Jurassic (163.5–168.3 Ma), echinoid cysts^12^; **(17)** France, Late Jurassic (157.3–163.5 Ma), crinoid and echinoid cysts; **(18)** Switzerland, Late Jurassic (157.3–163.5 Ma), echinoid cysts; **(19)** Israel, Middle Jurassic (163.5–166.1 Ma), echinoid cysts; **(20)** Dagestan, Turkmenistan and northern Caucasus, Middle Jurassic (163.5–166.1 Ma), echinoid cysts; (**21)** Germany, Holocene (Subboreal, 2.5–5 ka), spermatophores^22^,^23^; **(22)** Denmark and West Greenland, Quaternary (late glacial (10–12 ka) and postglacial (0.5–5 ka), respectively), egg sacs^19^; **(23)** northwestern Atlantic, Middle Miocene–Early Pleistocene (c. 0.78–15.97 Ma), eggs^16^; **(24)** Mexico, Gulf of California, Holocene, eggs^17^; **(25)** Brazil, Campos and Santos Basins, Early Cretaceous (100.5–113.0 Ma), eggs^24^,^25^,^26^; **(26)** Australia, New South Wales, Holocene (5.63 ka), eggs^15^,^18^; **(27)** Banda Sea, Late Pleistocene–Holocene, egg envelopes^20^,^21^. See ref. 14 for references dealing with echinoderms inferred to be infested by copepods resulting in cysts or swellings **(13–20)**. Image created in ArcGIS version 10.4.0.5524 (http://www.esri.com/software/arcgis/arcgis-for-desktop) using Light_Grey_Canvas basemap (which is Copyright: © 2015 Esri, Inc.; http://www.arcgis.com/home/item.html?id=8b3d38c0819547faa83f7b7aca80bd76) and Adobe Illustrator CS6, version 16.0.0 (32-bit) (http://www.adobe.com/uk/products/illustrator.html).

**Table 1:** Distribution of harpacticoid copepods in Lower Miocene amber samples from Campo La Granja, Chiapas, Mexico.

Harpacticoid taxa	Figures	Family	Number of specimens	Amber fragments (IHNFG no.)
*Cletocamptus* sp. (?)	Fig. 4e,g	Canthocamptidae	3	5311, 5312, 5313
*Enhydrosoma* sp. 1	Fig. 4h	Cletodidae	1	5314
*Enhydrosoma* sp. 2	Fig. 4j	Cletodidae	1	5314
*Enhydrosoma* sp. 3	Fig. 5b	Cletodidae	1	5314
Cletodidae sp.	Fig. 4d	Cletodidae	1	5315
*Darcythompsonia* sp.	Figs 4bl and 5i	Darcythompsoniidae	3	5312, 5315, An4
*Leptocaris* sp.	Fig. 5a,c,d,f,h	Darcythompsoniidae	35	4920, 4922, 5312, 5314, 5315, An1, An2, An3, An4
*Halectinosoma/Pseudobradya* sp.	Fig. 5j	Ectinosomatidae	1	4899
Ectinosomatidae sp. (?)	Fig. 5e	Ectinosomatidae (?)	1	5315
Laophontidae sp.	Fig. 4c	Laophontidae	1	4948
Unidentified adults/copepodids	Figs 4f,i,k and 5g	Indeterminable	21	4920, 5311, 5313, 5314, 5315, An1, An2, An4, Is1
